# Disturbance induced decoupling between host genetics and composition of the associated microbiome

**DOI:** 10.1186/1471-2180-13-252

**Published:** 2013-11-09

**Authors:** Karl Mathias Wegner, Nils Volkenborn, Hannes Peter, Alexander Eiler

**Affiliations:** 1 Helmholtz Centre for Polar and Marine Research, Alfred Wegener Institute, Coastal Ecology, Wadden Sea Station Sylt, Hafenstrasse 43, 25992, List/Sylt, Germany; 2 Department of Biological Sciences, University of South Carolina, 715 Sumter Street, Columbia, SC 29205, USA; 3 Benthic Ecology Laboratory, IFREMER, DYNECO, BP70, Plouzane 29280, France; 4 Laboratory of Aquatic Photobiology and Plankton Ecology, Institute of Ecology, University of Innsbruck, Technikerstrasse 25, Innsbruck 6020, Austria; 5 Department of Ecology and Genetcis, Uppsala University, Limnology, Norbyvägen 18D, Uppsala 75236, Sweden

**Keywords:** Microbiota, Population structure, Stress, Pathogen, Biological invasion, Pacific oyster, Crassostrea gigas, Vibrio, Mycoplasma

## Abstract

**Background:**

Studies of oyster microbiomes have revealed that a limited number of microbes, including pathogens, can dominate microbial communities in host tissues such as gills and gut. Much of the bacterial diversity however remains underexplored and unexplained, although environmental conditions and host genetics have been implicated. We used 454 next generation 16S rRNA amplicon sequencing of individually tagged PCR reactions to explore the diversity of bacterial communities in gill tissue of the invasive Pacific oyster *Crassostrea gigas* stemming from genetically differentiated beds under ambient outdoor conditions and after a multifaceted disturbance treatment imposing stress on the host.

**Results:**

While the gill associated microbial communities in oysters were dominated by few abundant taxa (i.e. *Sphingomonas, Mycoplasma*) the distribution of rare bacterial groups correlated to relatedness between the hosts under ambient conditions. Exposing the host to disturbance broke apart this relationship by removing rare phylotypes thereby reducing overall microbial diversity. Shifts in the microbiome composition in response to stress did not result in a net increase in genera known to contain potentially pathogenic strains.

**Conclusion:**

The decrease in microbial diversity and the disassociation between population genetic structure of the hosts and their associated microbiome suggest that disturbance (i.e. stress) may play a significant role for the assembly of the natural microbiome. Such community shifts may in turn also feed back on the course of disease and the occurrence of mass mortality events in oyster populations.

## Background

Emerging diseases of marine organisms often manifest in mass mortalities associated with environmental perturbations such as heat stress events [1]. This also applies to marine bivalves where infectious agents cause detrimental effects by profiting from increased temperatures in combination with a weakened immune response of the host [2,3]. Prominent examples for such mass mortalities are ‘summer mortalities’ of farmed and wild Pacific oysters *Crassostrea gigas* in several localities worldwide [4-6]. Here, the outcome of an infection is thought to be driven by a complex interplay of abiotic factors (e.g., temperature) and biotic factors (e.g., host genetic or immune system effects [7,8] and/or reproductive state [9]). More recently, host-associated microbiota have also been suggested to play an important role in determining host fitness [10,11]. Such effects can be mediated by providing additional energy sources by chemosynthesis [12] but also in defence against disease by either preventing establishment of pathogens or directly attacking them with antimicrobial effector molecules [13]. The use of probiotics in bivalve aquaculture has therefore been discussed as a means of preventing loss due to disease [14]. However, relatively little is known about microbial communities of native populations and their response to environmental perturbations.

Microbial communities residing in different organs of several oyster species have only recently been described by using molecular, culture independent techniques [15-17] that allow intra- and interspecies comparisons [18] and the exploration of environmental factors, such as temperature [19]. For example, oysters invading the Mediterranean from the Indian ocean maintained some of their associated microbes throughout the invasion process [18]. This is not self evident as biological invasions inevitably represent a drastic shift in abiotic and biotic conditions including the exposure to different microbes in the new environment.

Using next generation amplicon sequencing of individually tagged 16S rRNA-PCR reactions [20], we here assessed the combined effects of host population and disturbance/host stress on the microbial communities associated with gill tissue of Pacific oysters *Crassostrea gigas* stemming from populations only very recently invading the North Sea. The invasion of Pacific oysters into the Wadden Sea part of the North Sea originated from aquaculture activities in the 1990s [21], and today Pacific oysters locally represent the dominant epibenthic bivalve species [22]. Oyster populations in the northern and southern parts of the Wadden Sea stem from two genetically distinct invasion sources [23]. These separate invasions are also interesting in terms of summer mortality events because summer mortality has been observed only in southern populations so far [24].

Individual microbial communities can also be influenced by host genetics, either between populations (i.e. phylogeography and genetic differentiation) [25] or within populations (i.e. relatedness). Strong skew in reproductive success among individual breeders [26] is common in marine bivalves displaying high juvenile mortality (i.e. type III survivor curves) and can lead to increased genetic differentiation. In turn this can also lead to genetic differentiation on small spatial scales and therefore we here compare microbial communities in oysters from different reefs that most likely originated from different spatfall events. Our sampling scheme allowed us to evaluate the relative importance of host population genetic structure independent of confounding effects of geography. We investigated a total of 40 individual oyster microbiomes within three separate oyster reefs stemming from two tidal basins in the northern Wadden Sea. By exposing half of the oysters to a disturbance treatment, we tested if stress in combination with environmental change causes a shift in the microbial communities and if such a shift is associated with an increase in the abundances of potentially pathogenic bacteria during periods of stress. This could potentially reveal whether mortality events originate from environmental or intrinsic reservoirs and if such events are possibly associated with the demise of beneficial microbes.

The artificially induced microbial community shift can thus be used to compare reaction norms of microbial communities in naturally replicated host genotypes across genetically differentiated host populations. Our detailed objectives were 1) to test the differentiation of individual host-associated microbial communities according to population and individual genetic differentiation (i.e.phylogeography and relatedness, respectively) and 2) to identify bacterial taxa from those communities that respond similarly to disturbance stress across individuals and host populations in order to evaluate whether such pulsed perturbations suffice to promote a rise in opportunistic and potentially pathogenic strains.

## Methods

### Sampling & experimental procedures

**Figure 1:**
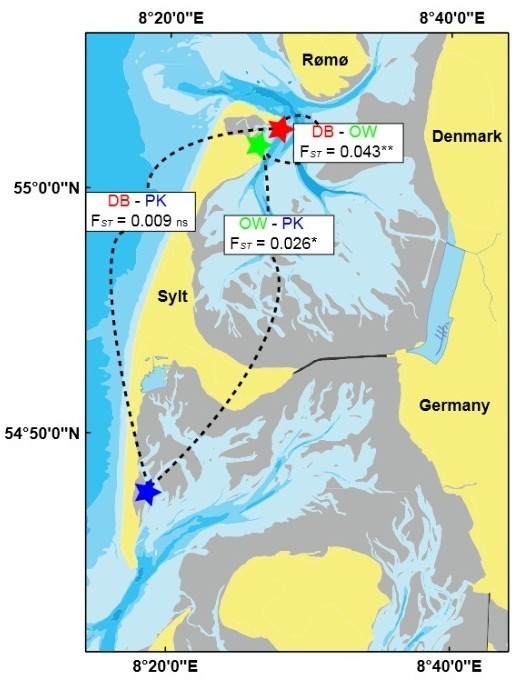
**Geographic location and genetic differentiation between investigated oyster beds.** Stars indicate the location of the oyster beds and boxes the pairwise genetic differentiation (F_*ST*_) between host populations. ** p < 0.001, * p < 0.01, ns: not significant.

To explore the relationship between host genetic differentiation and microbiome composition in response to environmental stress we collected oysters on 18^th^ and 23^rd^ of January 2008 from three oyster beds in the northern Wadden Sea covering two tidal basins, the Sylt-Rømø-Bight (Diedrichsenbank - DB 55° 02′ 32.13″ N, 08° 27′ 02.86″ E, Oddewatt OW 55° 01′ 41.20″ N, 08° 26′ 17.31″ E) and the Hörnum Deep (Puan Klent PK 54° 47′ 29.59″ N, 08° 18′ 18.52″ E, see Figure 1). We chose to collect oysters in winter because diversity and abundance of pathogenic strains are correlated with temperature [27] and the input of transient open water pathogens could potentially be minimised this way. From each bed we collected 20 oysters by picking single, unattached individuals from soft-bottom mud flats. After collection half of the oysters were frozen (−20°C) while the other half was transferred to large buckets (20 L) filled with sand-filtered seawater (salinity 29‰). We kept groups of oysters in these buckets under constant aeration at densities of 10 oyster/bucket. To minimise allochthonous input of microbes and facilitate spread of potential pathogens we decided to use static conditions with no flow-through and did not feed the oysters during the experimental treatment. All experimental animals were exposed to a heat-shock treatment by increasing water temperature from ambient 2°C to 26°C over a time span of 10 days, before individuals were frozen at −20°C. We chose this steep temperature increase to maximise heat-induced stress for the host and to allow potential pathogens to proliferate since temperatures of >20° are often associated with pathogen induced mass mortalities [24,28]. Our disturbance treatment thus combined aspects of transfer, food and heat stress. All experiments complied with German legal standards. For genetic analyses a small piece of gill tissue was removed from each individual oyster and DNA was extracted using the Wizard Genomic DNA Purification kit (Promega, Mannheim) following the manufacturer’s instructions. We decided to use gill tissue because gills constitute large contact surfaces to the surrounding water and should thus capture both, resident bacteria as well as bacteria from the environment. Furthermore, it has been shown that gill microbiota of Mediterranean oysters are more distinct from surrounding waters than those associated with gut tissue [18]. We used 14 oysters per bed (7 ambient ones frozen immediately and 7 exposed to disturbance treatment in the lab) for genetic analysis and microbiome sequencing.

### Genotyping of host microsatellite markers

To measure host population structure we amplified ten polymorphic microsatellite loci [29] covering nine linkage groups. These included Cg157 (LG I 141.5 cM), Cg_194 (LG I 28.7 cM), Cg136 (LG II), Cg193 (LG III), Cg_164 (LG IV), Cg173 (LG V), Cg_i28 (LGVI), Cgi24 (LG VII), Cg172 (LG IX) and Cg_181 (LG X) [30]. Loci were amplified using M13-tail labelling [31] in 20 μl volume using 4 μl of 5x concentrated buffer containing MgCl_2_ (Promega, Mannheim), 10 nM of each dNTP, 13.9 μl of H_2_O, 5 nM of each locus specific primer, 8 pmol of one M13-tail labelled with either FAM, VIC, NED or PET fluorophores and 1 unit of GoTaq Polymerase (Promega, Mannheim). Cycling used a standard protocol consisting of 5 min at 94°C followed by 28 cycles at 94°C (30 s) / 56°C (45 s) / 72°C (45 s). M13 tail incorporation was achieved in 8 cycles at 94°C (30 s) / 53°C (45 s) / 72°C (45 s), and a final extension at 72°C for 10 min. PCR products were pooled into sets of four loci with differently coloured labels and separated on a ABI Prism 3130 XL (LifeTechnologies, Darmstadt) capillary sequencer using a LIZ 500 size standard. Product sizes were scored using the GeneMarker v1.90 software (SoftGenetics) and pairwise genetic differentiation between populations was calculated as Wright’s fixation indices (F_ST_) according to Weir & Cockerham [32]. Pacific oysters are known to harbour substantial amounts of null alleles [33] that could bias any estimate of population differentiation. We therefore estimated the frequency of null alleles within our sample using the software freeNA [34]. Frequencies of null alleles were small for all loci and populations (range: 0 – 0.06) except for locus Cgi-194 where estimates were higher within all oyster beds (range: 0.05 – 0.15). We therefore excluded this locus from the analysis, and only report the corrected F_*ST*_ values after removal of loci with high frequencies of null alleles in all populations. Genetic distance between individuals was calculated as geometric AMOVA distances:

$$\partial^{2}=\sum_{s=1}^{S}{\left(p_{\mathrm{sj}}-p_{\mathrm{sk}}\right)}^{2}$$

[35],

where distances between individual genotypes *j,k* are summed over *S* loci. Calculations were performed using the R package *Gstudio*.

### Amplicon sequencing of microbial communities

Microbial diversity and composition was measured within a standardised amount of genomic DNA (30 ng). We used an informative part of the 16S rRNA gene spanning position 535-904/912 in the *E. coli* genome covering the variable regions V3 and V4 for ribotyping. Using a PCR product of this length increases the precision of taxonomic assignment [36] and should provide high resolution due to the inclusion of two variable regions. Initial testing of these primers revealed that they preferentially amplified host 18S rRNA (24 out of 24 randomly picked clones). To avoid cross-amplification we performed a nested PCR starting with primers specific for Eubacteria (27f: AGA GTT TGA TCA TGG CTC AG and 1492r: GGT TAC CTT GTT ACG ACT T). The first nested PCR consisted of 30 ng of genomic DNA, 0.05 μl of Hot start taq (5 unit/μl, Promega), 1 mM of each dNTP, 4 μl of reaction buffer (Promega), 1 μl of each forward and reverse primers (5 μM) and 11.5 μl of molecular grade water. Cycling started with an initial denaturation and hot start activation of 10 min at 95°C followed by a low number of 16 cycles of 30 s denaturation at 95°C, 30 s at 50°C and 90 s at 72°C and a final extension of 10 min at 72°C. One μl of each PCR product was then diluted in 99 μl of molecular grade water before the internal stretch was amplified for 454 sequencing. Here, each individual microbiome was tagged by a unique combination of multiplex identifiers (MID, Roche, Basel, CH) integrated into forward and reverse primers [37,38]. We used a total of 20 tagged primers consisting of the Titanium B sequencing adaptor (Roche, Basel), the 454 sequencing key, a MID tag and the gene-specific sequence. Hence, an example of a forward primer would have the following sequence: 5′-CCATCTCATCCCTGCGTGTCTCCGAC *TCAG***ACGAGTGCGT** CCACGAGCCGCGGTAAT -3′ and a reverse primer: 5′-CCTATCCCCTGTGTGCCTTGGCAGTCTCAG *TCAG***ACGAGTGCGT** CCGTCAATTCMTTTAAGTTT-3′, with the 454 sequencing key in italics, the MID tag in bold and gene specific sequence underlined. Combinations of forward and reverse MIDs were random with respect to treatment and oyster bed. Therefore any amplification bias introduced by the MID will be randomly distributed among groups.

After amplification single PCR reactions were purified using the MinElute 96 kit (Qiagen, Hilden) before 2 μl of each elution was used for pooling. To eliminate remaining primer-dimer both pools were purified again using Wizard PCR clean-up system (Promega, Mannheim) following the manufacturer’s instructions. After confirming the sole presence of the desired PCR product without any traces of primer by gel electrophoresis, the pool of individually barcoded PCR reactions were sequenced on the 454 FLX genome sequencer (Roche, Basel, CH) using Titanium chemistry. Sequencing was performed by GATC Biotech (Konstanz, Germany).

### Data analysis

Assignment of reads to individual PCRs was done using modified python scripts from the cogent package. In short, within each raw read we looked for the presence of both primers ensuring complete sequencing of the PCR product. Afterwards, we identified individuals by determining combinations of MID tags allowing for a maximum hemming distance of one in each MID tag. After correct assignment of single reads to an individual oysters, we used the AmpliconNoise pipeline [39] to remove pyrosequencing and PCR noise and Perseus to remove chimeric sequences using default parameters except for alpha and beta values for false discovery detection in Perseus, which were set to −7.5 and 0.5, respectively. Reads were trimmed by cutting off their forward and reverse primers.

We used scripts from the Qiime package [40] for the analysis of microbial diversity. In detail, we clustered sequences into operational taxonomic units (OTUs) using UCLUST using unique and cleaned reads assigned to individual oysters. Taxonomic assignment of OTUs found for each individual oyster was done using the naïve Bayesian Classifier [41]. We used an assignment certainty threshold of 60% for each taxonomic classification. As singleton reads overestimate the contribution of rare phylotypes [42] we removed singleton reads. All analyses were then based on the resulting OTU table to account for small strain specific differences and was used to calculate observed bacterial diversity (Shannon’s H’). Sufficient sampling of observed diversity was confirmed by rarefactions based on group specific microbiomes. Potentially pathogenic OTUs were identified by genus classifications and pooled according to genus affiliation. We used previously described genera of pathogenic bacteria in shellfish [3] and other marine organisms [43] to identify such potentially pathogenic bacteria. These included *Arcobacter* spp., *Citrobacter* spp., *Corynebacterium* spp., *Escherichia* spp., *Halomonas* spp., *Micrococcus* spp., *Mycoplasma* spp., *Photobacterium* spp., *Pseudoalteromonas* spp., *Pseudomonas* spp., *Shewanella* spp., *Staphylococcus* spp., *Streptococcus* spp., *Tenacibaculum* spp.. We used non-metric multidimensional scaling from the *vegan* R package to visualise distance matrices (Horn-Morisita distances, Wisconsin double square root transformation) between individual microbiomes. Statistical differences between treatments and oyster beds were analysed by means of multivariate permutational ANOVA (*adonis* function, Horn-Morisita distances) and comparisons between distance matrices were based on non-parametric Mantel tests or procrustes rotations of ordinations. To account for differences in sequencing depth between libraries we also resampled all communities to the lowest coverage using the perl script daisychopper (available at http://www.genomics.ceh.ac.uk/GeneSwytch/Tools.html). To further account for differences in library size, analyses relying on the abundance of OTUs (e.g. abundance - occupancy analyses) were based on relative abundances of ln-transformed read numbers within each oyster. All analyses were performed in R [44].

## Results

### Host genetic differentiation

We found significant genetic differentiation (F_*ST*_) in two out of the three pairwise comparisons between oyster beds (Figure 1). Interestingly with a F_*ST*_-value of 0.043 (P < 0.001) the largest pairwise differentiation was observed between the two oyster beds found closest to each other, i.e. Diedrichsenbank (DB) and Oddewatt (OW, geographic distance 2.5 km) while the genetic differentiation to a different tidal basin was lower (OW-PK: F_*ST*_ = 0.026, P = 0.002) or not even significant (DB-PK: F_*ST*_ = 0.009, P = 0.124, Figure 1). Similar results were obtained using individual genetic differentiation based on AMOVA distances and multivariate Permanova of the resulting distance matrix, with genetic variation among populations explaining 11.9% of the overall variation (P < 0.001 based on 1000 permutations). In concordance with the expectation of random sampling before treatment assignment we found no significant difference between “ambient” and “disturbed” oysters in terms of their genetic variation (R^2^ = 0.031, P = 0.159 based on 1000 permutations) and no significant interaction effect (R^2^ = 0.053, P = 0.257 based on 1000 permutations). Due to high within locus polymorphism the majority of variation was found among individuals (R^2^ = 0.797).

### Microbial communities of oysters before and after disturbance

**Figure 2:**
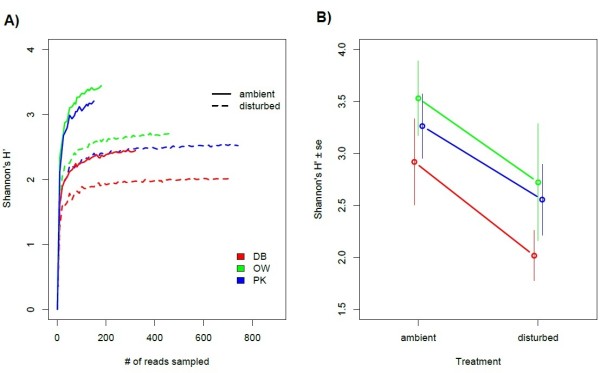
**Bacterial diversity (Shannon’s H’) of oyster gill microbiota stemming from different oyster beds. A)** Rarefaction curves of Shannon’s H’ in different oyster beds under ambient field conditions and after disturbance. Shown are rarefied means for treatment and origin groups from 10 resamples with a maximum number corresponding to the lowest coverage of a single microbiome in each group. Solid lines represent ambient conditions and dashed lines disturbed microbial communities. **B)** Observed values of Shannon’s H’ for individual oysters stemming from different oyster beds (mean ± se) showing significant differences between oyster beds (F_2,36_ = 3.55, P = 0.039) and a significant decrease of diversity after disturbance (F_1,36_ = 7.52, P = 0.009).

Out of the 52,092 reads that could successfully be assigned to an amplicon library for each individual, 38,029 reads passed our quality selection and de-noising criteria for further analysis. The resulting average library size per individual was 825 ± 80. With a total number of 4,464 unique operational taxonomic units (OTUs) distributed over 213 genera, microbial species richness was very high. However, only few OTUs occurred frequently and most OTUs occurred rarely (<1% within whole data set). After rigorous de-noising of our sequencing data we potentially underestimated species richness of the respective communities, but we could reliably calculate diversity (Shannon’s H’) for most experimental groups (Figure 2A). Microbial diversity was significantly lower in oysters originating from DB (GLM, F_2,36_ = 3.55, P = 0.039) especially under ambient conditions (Figure 2A,B). The disturbance treatment led to a significant decrease of bacterial diversity in oysters from all beds (Figure 2B, disturbance: GLM F_1,36_ = 7.52, P = 0.009, disturbance × oyster bed interaction: F_2,36_ = 0.80, P = 0.456).

**Figure 3:**
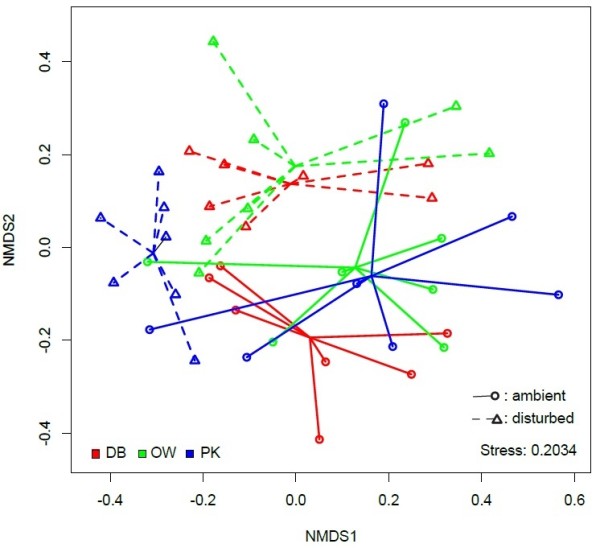
**Non-metric multidimensional scaling of bacterial communities associated with oyster gill tissue.** Ordination was based on Horn-Morisita distances from unique OTUs after Wisconsin double standardisation and square root transformation. Symbols show communities of single oysters with circles representing ambient communities and triangles representing disturbed communities. Solid and dashed lines connect single communities with group centroids. Colours code for different oyster beds.

Non-metric multidimensional scaling of the full bacterial communities from individual oysters suggested that communities were differentiated by treatment along both axes (Figure 3), which was confirmed by Permanova (effect of disturbance: R^2^ = 0.077, P = 0.006). Clustering of ambient group centroids in the ordination suggests that initially there was no significant difference between beds and large variation within beds under ambient conditions (Figure 3, Permanova, effect of oyster bed: R^2^ = 0.058, P = 0.211). Community shifts in response to the disturbance treatment however seemed to depend on the initial community composition and were differentiated in the NMDS as oysters from PK shifted vertically, while shifts occurred mainly horizontally for oysters stemming from OW and DB. This effect was however only marginally significant in the overall analysis (Permanova, disturbance × oyster bed interaction: R^2^ = 0.076, P = 0.073). Similar results were obtained with rarefied communities (n = 245 reads per library, disturbance effect: R^2^ = 0.078, P = 0.009, oyster bed effect: R^2^ = 0.054, P = 0.244, disturbance x bed interaction: R^2^ = 0.076, P = 0.081).

**Figure 4:**
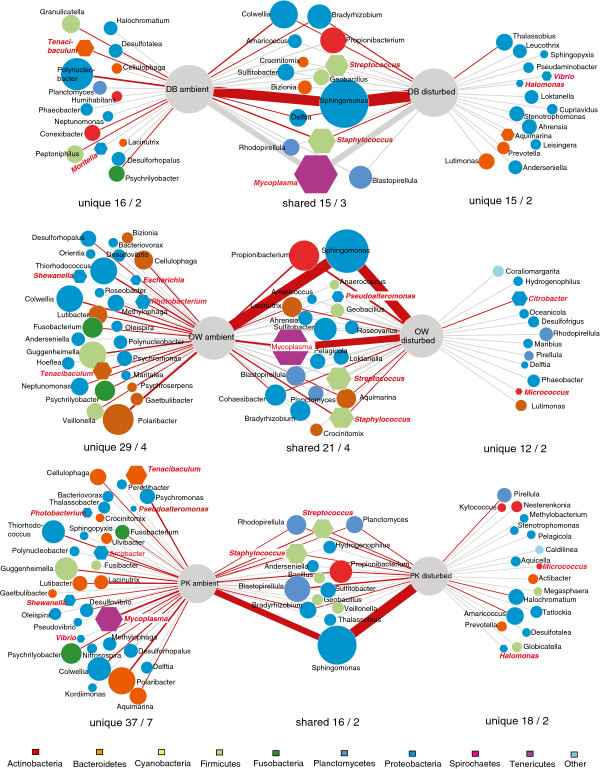
**Association network of bacterial taxa (genus level) in ambient and disturbance treatments of the three different oyster beds (DB, OW, PK).** Taxa are shown as circles with colour-coded phylogenetic relationship and size reflecting overall relative abundance (ln(x + 1) transformed) from a rarefied resampled data set. Lines indicate the occurrence in the respective treatment. Hence, taxa only related to one treatment occurred exclusively in either ambient or disturbed oysters while taxa related to both treatments occurred before and after the disturbance. Width of lines corresponds to the proportion of each taxon within each treatment. Red edges indicate significant distribution bias towards one treatment group. Hexagons represent genera known to contain pathogenic strains (printed in bold) and numbers below each group give the number of genera followed by the number of potential pathogens.

Proteobacteria represented the numerically most abundant phylum in both ambient and disturbed conditions (Figure 4). Numerical abundance of Proteobacteria was owed to the fact that the overwhelming majority of OTUs were affiliated with the genus *Sphingomonas* (30.6 – 64.1% for each treatment group, Figure 4) and only few other taxa reached comparably high numbers (e.g. *Flavobacteria* (*Bacteroidetes*)). Several taxa were characteristic for specific oyster beds or shifts during disturbance treatment (Figure 4). *Flavobacteria* (*Bacteroidetes*), for example, were common in OW and PK in ambient conditions rare in DB. All beds contained several genera unique to each treatment with ambient communities having higher proportions of unique taxa reflecting higher overall diversity. Disturbed communities from DB and OW oysters were shaped by OTUs associated with the genus *Mycoplasma* (*Tenericutes*) while *Planctomycetales* were characteristic for disturbed communities of PK oysters (Figure 4).

Contrary to our expectation we did not observe a significant increase in the proportion of reads containing potentially pathogenic bacterial genera after the disturbance treatment (paired *t*-test, t = 0.990, df = 17, P = 0.336) nor did we find an increase in their taxonomic abundance (DB: 2 taxa unique in ambient communities vs. 2 taxa in disturbed communities, OW: 4 vs. 2, PK: 7 vs. 2, Figure 4). While the overall load of genera containing known pathogenic strains did not change significantly, single genera increased or decreased strongly in response to the disturbance (Figure 4). Reads classified as *Mycoplasma* increased strongly in abundance while other well established shellfish pathogens like *Vibrio* were very rare (Figure 4, frequency 0.013%).

**Figure 5:**
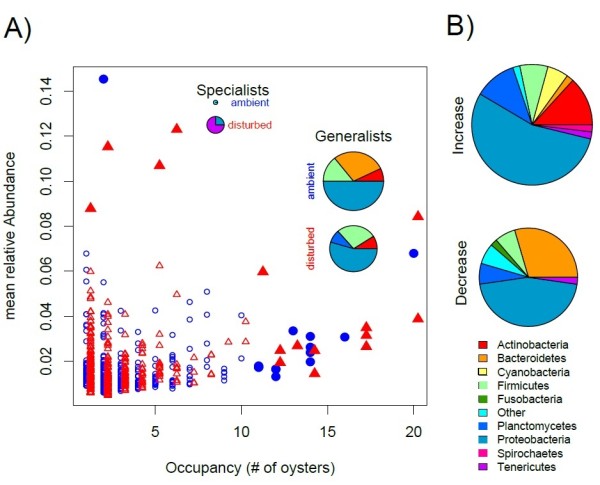
**Relationships between abundance and occupancy of OTUs recovered from oyster gill tissue. A)** Abundance occupancy plot showing the relative mean abundance ((ln + 1) transformed) of each OTU as a function of occupancy (i.e., from how many oysters it was recovered) for ambient (blue circles) and disturbed conditions (red triangles). Filled symbols mark generalists (abundance less than 1% in more 50% of oysters) and specialist (highly abundant in few oysters) OTUs. Pie charts show the taxonomic affiliation of generalists and specialists, where the size of the pie corresponds to the number of OTUs. **B)** Taxonomic composition of all taxa that increased (upper panel) or decreased (lower panel) in abundance and occupancy. Pie size represents number of OTUs found in each group and colours code for different phyla.

Abundance (i.e., how frequent an OTU occurs in a host) is often positively correlated to occupancy (i.e. the number of hosts an OTU is observed in) [45]. We found such a significant relationship between the mean relative abundance of OTUs in single oysters and the number of oysters they occurred in (occupancy) only after disturbance (Spearman’s rank correlation: ρ = 0.175, P < 0.001) while ambient bacterial communities did not show such a relationship (Spearman’s rank correlation: ρ = −0.004, P = 0.931). In both environments we could identify some generalist taxa (moderately abundant in more than 50% of hosts [46,47]). Specialist taxa (highly abundant in less than 25% of hosts) were rare under ambient conditions but we could observe a shift towards increased specialisation in disturbed communities that was mainly associated with a steep increase in relative abundance of OTUs associated to the genus *Mycoplasma* (Figure 5A).

Overall, only few OTUs were observed in both treatments (n = 298 corresponding to 6.7%) and we could observe a net increase in relative OTU abundance (paired *t*-test, mean difference = 0.19, t = 3.96, df = 297, P < 0.001) but a net decrease in occupancy (paired *t*-test, mean difference = −0.32, t = −2.19, df = 297, P = 0.029). Taxonomic differences between ambient and disturbed communities were mainly associated to a decrease of *Bacteroidetes* (especially *Flavobacteria*) and the increase of *Actinobacteria* in disturbed oysters (Figures 4 and 5B), which corresponded to the disappearance of generalist *Flavobacteria* after disturbance (Figure 5A).

### Correlation of microbial community and host population genetic structure

In contrast to host population structure (Figure 1) we did not find a significant difference in microbial community structure on the level of oyster beds (Figure 3). Considering that most genetic as well as microbial community variation was partitioned between individuals, microbial communities could also associate with individual genotypes within populations rather than with geographically and genetically separated host populations. Accordingly we found a significant correlation of individual pairwise genetic distances (AMOVA) and microbial community distances (Bray-Curtis dissimilarity) for ambient oysters using non-parametric Spearman’s rank correlation reflecting the non-normal distribution of microbial community distances (Mantel test: R = 0.137, P = 0.045). This result was supported by a correlation of symmetric procrustes rotations of both ordinations (R = 0.48, P = 0.018 based on 1000 permutations). Such a result was not observed for disturbed oysters (Mantel test: R = −0.07, P = 0.756, Procrustes rotation R = 0.19, P = 0.714 based on 1000 permutations) indicating that original communities may have adjusted to different host genotypes while these association broke apart as a result of disturbance.

**Figure 6:**
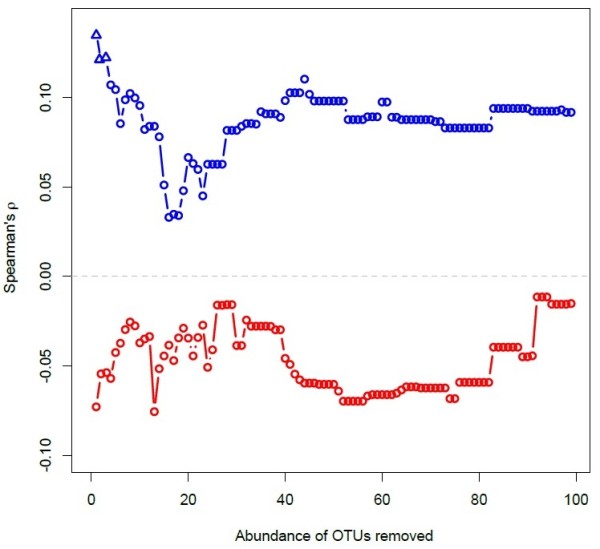
**Correlation coefficients (Spearman’s) between genetic distance among individuals and similarity of microbial communities associated with host gill tissue.** The blue and red lines represent ambient and disturbed communities, respectively. OTUs were iteratively removed with increasing abundance thresholds and significance of each correlation was assessed by Mantel tests with 1000 randomisations. Significant correlations (p < 0.05) are shown as triangles and could only be observed for correlations containing rare parts of the ambient communities.

We subsequently tested whether rare or common components of the bacterial communities were responsible for the observed correlation and removed OTUs in a sliding window approach based on their abundance. In detail, we first removed OTUs that occurred only twice in the data set and repeated the correlation analysis for both ambient and disturbed oysters. This procedure was iterated with increasing abundance cut-off values up to an abundance threshold of 100, which represents a reasonable upper limit because communities contained only few taxa after this procedure and only changed little with higher thresholds. We only found significant positive correlations for communities containing rare OTUs (overall abundance threshold 2–4) while all disturbed communities correlated negatively with genetic distance among individuals (Figure 6).

## Discussion

### Linkages between microbial communities and host genetics

Using a barcoded 16S rRNA 454 amplicon sequencing approach, we showed that the undisturbed ambient microbial communities associated with gill tissue of invasive Pacific oysters *Crassostrea gigas* from three genetically differentiated reefs did not follow genetic differentiation of the host populations, although environmental variation and host population structure have previously been implicated in the composition of gut microbial communities [25]. Oyster gill microbiota, on the other hand, harboured a substantial amount of variation between individuals (Figures 2 and 3). The between individual variation in microbial community composition correlated with genetic relatedness of the oysters, suggesting that microbial communities might assemble according to individual hosts or even host genotypes. Stable host associations have been reported for several gut microbiota in a variety of host species [48-51]. The human gut bacterial community, for example, is considered to be stable over extended periods of time, but is also unique for each individual [51] and similar between related individuals [52]. Similarly, stable associations have been reported from insects [50] and crustaceans [49] and have also been observed in oyster species in the Mediterranean where associations were stable even after invasion from the Red Sea [18]. Such stable associations harbour an environmental component depending on food [49] but also genetic components as suggested by similar communities found within mother-twin triplets [53]. The fact that the similarity in microbial communities correlated with the genetic relatedness of the Pacific oyster demonstrated here, further suggests that bacterial communities are not only unique to individuals but can also assemble according to host genotypes. In combination with the lack of significant differentiation of community structure between oyster beds this suggests that larger scale environmental differences between beds may play a limited role when compared to host genotype. Furthermore, correlations between genetic microbial community distances depended to a large degree on OTUs only occurring rarely in the communities (Figure 6). This suggests that while abundant taxa may lead a generalist life style and are found in the majority of host genotypes, rare specialists within the community assemble according to host genotypes. An alternative explanation for the formation of genotype specific microbiome associations is vertical inheritance [54,55]. While we cannot rule out this possibility for Pacific oysters, the transient nature of the genotype specific associations suggests that previously encountered disturbance events should also have led to the loss of the inherited genotype-specific microbiota. A recovery of genotype specific associations prior to our experiment therefore rather suggests an uptake from the environment.

Genetic and phenotypic variation among populations in marine invertebrates with type III survival curves can occur because of uneven reproductive success among individual breeders (i.e., sweepstakes reproductive success [56]). Bias in reproductive success between spawning events potentially led to the genetic differentiation of the investigated oyster beds (Figure 1). Given that we did not find patterns of genetic differentiation compatible with a stepping stone model or with distance-dependent gene flow among oyster beds, a successive formation of oyster beds from genetically differentiated spatfall events in time is more likely. Successive waves of settlement from genetically different broodstocks will also lead to structure within beds and increase the genetic diversity within populations.

Sweepstakes reproductive success can also lead to linkage disequilibrium, because a reduced effective population size will amplify the effect of genetic drift and thus create an overrepresentation of certain allelic combinations within haplotypes [57]. This also applies to linkage disequilibrium between selectively neutral markers (in this case microsatellites) and genes with functional relevance, thus representing potential targets of selection. The genetic differentiation that we found between populations as well as between individuals should therefore be interpreted as a marker for different spatfall events where variation in functional genes (e.g. immune genes) involved in microbial colonisation can influence the observed association of host genetics - microbiota relationships.

### Disturbance of microbial communities in oyster gills

With our parallel tag-sequencing approach we were able to describe the microbial communities associated with Pacific oyster gill tissue in unprecedented detail, yet the 38,029 reads used in this analysis were not sufficient to capture the total taxonomic richness present in single oysters. This represents the typical picture found in marine microbial communities in general [20] as well as in sediment and open water communities from the same habitat [58]. The strongly skewed negative binomial distribution of OTUs suggests however that the taxonomic resolution was sufficient to reliably estimate bacterial alpha diversity expressed as Shannon’s H’ (Figure 2A). Additionally, the parallel characterization of microbial diversity in a high number of individuals from different oyster beds offers a high level of detail and biological replication. Previous studies on the characterisation of microbial communities associated with oyster species have either been focused on a cultivatable subgroup of bacteria [5,59] or used techniques of lower taxonomic resolution [18] or coverage [15,16,60] and only very recently pyrosequencing approaches have been used to characterize microbiota of oysters [17]. The gill microbial communities in our study were dominated by OTUs affiliated to the α-proteobacterial genus of *Sphingomonas* sp. The α-proteobacteria are dominant in the open water of the Wadden Sea, but rather belong to the SAR11 group [58]. While we also discovered SAR11/Pelagibacter in ambient samples of DB, its overall abundance was very low, indicating that the host associated microbiota is not an exact mirror image of the environment. This is also reflected in gill associated microbial communities of other oyster species that differ more strongly from the surrounding sea water than for example gut communities [18]. The numerical abundance of α-proteobacteria in open water could however partly been attributed to PCR bias by preferential amplification of sequences from this taxonomic group [61]. The dominant genus detected, was *Sphingomonas* which contains opportunistic species [62] and can also commonly be found in gill tissue of European plaice *Pleuronectes platessa* from the same region [38]. It was also abundant on freshly prepared cod in Iceland [63], indicating that this genus can reach high numbers on living hosts but is quickly outcompeted after the host’s death.

Dominance of a few closely related OTUs has been reported for other species of oysters. Zurel et al. [18] for example found that between 59 – 79% of OTUs in *Chama* spp. oysters in the Red Sea and the Mediterranean belonged to OTUs from the class *Oceanospirialles* closely related to the genera *Spongiobacter* or *Endozoicomonas (Hahellaceae),* which is known for symbiotic associations. While we also observed 47 OTUs from the *Oceanospirialles,* these were relatively rare (99 reads in total) and only a single OTU was affiliated to the family *Hahellaceae.* Similarly, we only found very few OTUs classified as *Arcobacter* spp. (13 OTUs, 16 reads), which represent a major and common component of Chilean oysters *Tiostrea chilensis*[60]. This suggests that oyster microbiomes can have similar structures in terms of abundances but dominant taxa differ strongly between species, habitats and sampled tissues. Under certain environmental conditions gut communities of other *Crassostrea* species were found to be dominated by *Mycoplasma*[17], which also became dominant in some oysters after disturbance in our experiments (Figure 5A). The natural dominance of *Mycoplasma* in oysters from much warmer habitats [17] may thus suggest that *Mycoplasma* represents a temperature sensitive part of oyster microbiota and may proliferate preferentially at higher temperatures.

Host stress and abiotic disturbance both could have contributed to the major shift in microbial community structure (Figure 3). The direction and magnitude of the shift was dependent on the initial community composition, and although no significant differences were observed between oyster beds in ambient conditions there was some indication for oyster bed specific shifts (Figures 3 and 4). The strongest shifts occurred in the beds with initially high microbial diversity (OW and PK), manifested in a sharp decrease in microbial diversity. In the oyster bed with low diversity on the other hand we observed no significant change in bacterial diversity (Figure 2). Our disturbance treatment represented a drastic change in abiotic conditions and probably resulted in multifaceted host stress responses as well as community shifts. We cannot disentangle what component of stress (food, transfer, or heat stress) or microbial community response caused the observed shifts. Our aim was however to compare the undisturbed natural community to a disturbed community in stressed hosts under conditions that can facilitate disease outbreaks (i.e., heat waves, food depletion, accumulation of waste products). We could not observe an overall net increase of obvious pathogen candidates like *Vibrio*[5,59]. Only OTUs affiliated to Mycoplasma, which can cause disease in shellfish [3], showed a strong increase in disturbed communities (Figure 4). *Mycoplasma* were also found to dominate microbialcommunities in the gut of Eastern oysters *Crassostrea virginica*[17]. However, since genus affiliation will not be sufficient to reliably identify pathogenic strains, controlled infection experiments are needed to evaluate the true pathogenic potential of the strains detected here. Furthermore, since we could neither invoke disease nor observe an increase in the abundance or occurrence it seems unlikely that disease agents are a constitutive part of the oyster microbiome, suggesting that disease outbreaks arise from environmental sources.

*Mycoplasma* was also the taxon that showed the strongest shift towards a specialist lifestyle (highly abundant in few hosts, [46,47], Figure 5A) and mainly drove the trend for higher abundances of specialist taxa in oysters exposed to disturbance. This shift towards higher degrees of specialisation also resulted in a positive relationship between the number of oysters hosting a specific OTU (i.e., occupancy) and the mean relative abundance of the respective OTU, which was absent from the ambient communities (Figure 5A). Such a positive relationship between abundance and occupancy is the null-expectation [45] and its absence under ambient conditions can probably be attributed to the frequent occurrence of rare taxa assembling in a genotype specific manner. On the other hand, only a small subset of OTUs shared between treatments were actually spreading and increasing in abundance (mainly *Actinobacteria*, *Sphingomonas* and *Mycoplasma*) while others got selectively lost in stressed oysters (mainly *Flavobacteria*).

## Conclusion

In winter months the microbiome in gill tissue of the invasive Pacific oyster, *Crassostrea gigas*, is dominated by few highly abundant taxa but show a high taxonomic diversity with many rare taxa supporting previous observations from microbial communities in marine sediments [20,58]. The β-diversity of natural, ambient communities correlated with individual host relatedness rather than with genetic differentiation between oyster beds suggesting that communities are stable within individuals [18,51] and that rare species are associated with genetic differentiation of the host. This association was lost when the host was stressed by our disturbance treatment (Figure 6). The subsequent shift in microbial community structure was driven by a loss of rare bacterial strains and an increase in the abundances of specialist strains. This suggests that genotype-specific associations are the result of the overall community diversity including rare phylotypes. If it is true that the disturbance of ambient host genotype – microbial community associations are an important component in the defence against infections, it will be very difficult to control disease by for example administering probiotics. Therefore, monitoring microbial communities during an actual infection will be an important future avenue of research to address the role of genotype specific microbial communities for host fitness and to improve our ability to predict mass mortality events in benthic populations.

## Availability of supporting data

Data are available at http://dx.doi.org/10.1594/PANGAEA.819896

## Competing interests

The authors declare that they have no competing interests.

## Authors’ contributions

KMW planned the research, performed molecular labwork, and led the writing of the manuscript, NV conducted the experimental field and lab work, data analyses was done by KMW, HP and AE. All authors read and approved the final manuscript.
